# Effect of Uniaxial Fatigue Aging and Fabric Orientation on Low Impact Velocity Response of Glass Fibers/Elium Acrylic Composite Laminates

**DOI:** 10.3390/ma14154089

**Published:** 2021-07-22

**Authors:** Tomasz Libura, Rodrigue Matadi Boumbimba, Alexis Rusinek, Zbigniew L. Kowalewski, Tadeusz Szymczak, Pierre Gerard

**Affiliations:** 1 Institute of Fundamental Technological Research of the Polish Academy of Sciences, Pawińskiego 5B, 02-106 Warsaw, Poland; zkowalew@ippt.pan.pl; 2 LEM3, Arts et Métiers ParisTech, Université de Lorraine, CNRS, F-57000 Metz, France; rodrigue.matadi-boumbimba@univ-lorraine.fr (R.M.B.); alexis.rusinek@univ-lorraine.fr (A.R.); 3 Motor Transport Institute, Jagiellonska 80, 03-301 Warsaw, Poland; tadeusz.szymczak@its.waw.pl; 4 ARKEMA, Groupement de Recherche de Lacq, F-64170 Lacq, France; pierre.gerard@arkema.com

**Keywords:** Elium acrylic, glass fibers, composite laminates, fatigue aging, low impact velocity, damage

## Abstract

Impact resistance is one of the most critical features of composite structures, and therefore, its examination for a new material has a fundamental importance. This paper is devoted to the characterization of the fully recyclable thermoplastic ELIUM acrylic resin reinforced by glass fabric woven, which belongs to a new category of materials requiring advanced testing before their application in responsible elements of engineering structures. Its high strength, low weight as well as low production cost give excellent opportunities for its wide application in the automotive industry as a replacement of the thermoset-based laminates. The study presents an experimental work concerning the effect of damage due to low and high cyclic fatigue aging of two groups of specimens, first with the woven fabric orientations of [0°/90°]_4_ and secondly with [45°/45°]_4_, on the low impact velocity properties. The impact resistance was measured in terms of load peak, absorbed energy, penetration threshold and damage analysis. The low velocity impact results indicate that the uniaxial cyclic loading (fatigue aging) of the material leads to the reduction of impact resistance, especially at the high impact energy levels. Scanning Electron Microscopy (SEM) and Computed Tomography (CT) scan observations reveal that the damage area grows with the increase of both strain amplitude and impact energy.

## 1. Introduction

Nowadays, automobile manufacturers try to build cars using novel materials that are more lightweight, stronger and cheaper. For this purpose, plastic elements have been introduced to the construction of vehicles, as well as thermosetting resins based on the laminate composites, which are 30% and 50% lighter than steel and aluminum alloy [1], respectively. Feldmann evaluated in 2008 [2], that the metal elimination from the construction of cars would increase a demand for energy by 26%. Therefore, the usage of polymers is necessary, as the average weight of the European cars increases continuously. For example in 1995, the average mass of car was 950 kg, while today it is around 1200–1350 kg [2].

Due to the newest European emission standards of carbon dioxide and current recycling requirements, the usage of organic matrix composites in the transportation industry has increased considerably. In addition to the weight reduction of single elements and entire machines or constructions, their application offers many simple adaptations in the complex structural elements [3]. In order to meet the current requirements, a new reinforced laminate with fully recyclable Elium acrylic thermoplastic resin has begun to play an increasingly important role [2,4]. Among the basic advantages of thermoplastic liquid resins application, one can indicate their compatibility with a large panel of existing ancillaries, mold construction and mixing machines for composites structures that reduces the cost of their usage significantly.

The acrylic based composites have attracted many branches of the industry recently. Therefore, a large number of studies are focused on the processing, characterization of elastic properties and low velocity impact resistance. Some basic features of composites are already known, as they have been determined by many researchers. Boufaida et al. [5] and Kinvi-Dossou et al. [6] conducted classical macroscopic mechanical tests, e.g., tensile, bending and shear tests (on both intralaminar and interlaminar composites), and revealed that the glass fibers reinforced Elium (GFRE) composite has a higher structural toughness then that for the thermoset-based composites observed, leading to its wider industrial applications. Obande et al. [7] and Cadieu et al. [8] evaluated the viscoelastic and impact features of the GFRE composites, containing Elium 150. Matadi Boumbimba et al. [9] tested the hardened composite plates by adding different amounts of the acrylic tri-block copolymers (Nanostrength^®^). It was subjected to the low impact velocity tests under different levels of the impact energy and temperature. The results exhibited that the structural response of the newly developed thermoplastic structures is highly dependent on the microstructure of the material as well as on the interface between both components. The impact results at different levels of energy and temperature showed a clear stress sensitivity effect of Elium microstructure taking the viscoelastic character until a given stress threshold was achieved, above which damage developed. Detailed fracture and damage analysis of the impacted specimens has proven that the effect of loading rate on the structural response of glass/Elium (stiffness, dissipated energy) is comparable to that for the non-recyclable thermosetting composites observed. However, it is well know that material properties can be also affected by damage and fracture kinetics which often occur during exploitation of structures. Therefore, in order to reflect how the long term mechanical processes (which depend on the working conditions) may change material properties, a selected loading histories should be taken into account. As a consequence, in 2018 Freund et al. studied the exposition of the material in question to moisture cycles [10]. The results allowed the distinguishing of two aging effects: the composite yielding induced by the presence of water molecules within the polymer network and the structural damage by weakening the fiber/matrix interface. In turn, the effect of temperature and moisture content on the damage scenario in an acrylic-matrix and GFRE composite under quasi-static tensile loading was studied by Boissin et al. [11]. The authors presented how large the influence of those factors were and how they affect the relationship between transverse cracks in composite and working conditions. In the present work the fatigue aging was selected as the process that can affect the internal structure of the GFRE composite during impact.

Fatigue leads to the progressive, localized and permanent structural damage that occurs in materials subjected to cyclic stresses and strains appearing during exploitation. Usually, it may result in cracks initiation, their propagation and finally to a fracture after a sufficient number of repetitive loading cycles. It may produce a failure, which occurs at the stress level much lower than the yield stress of the material tested. An importance of the fatigue process has been discovered firstly by Wöhler (1860), who concluded that the cyclic stress range is more important than the peak stress and introduced the concept of endurance limit (cyclic fatigue limit). Damage evolution under fatigue of the laminated composites is different than that in metals observed. The composites belong to the specific group of materials that can endure some assumed prime stiffness loss (fatigue damage) over its application [12,13]. The previous studies on fatigue tests [14,15] conducted on the laminated composites indicated that the plastic deformation is one of the most significant features of matrix-dominated composites. Generally, three basic steps of the fatigue damage can be distinguished: (i) crack initiation; (ii) crack growth, and finally (iii) ultimate failure. In the composite with the warps (longitudinal yarns) oriented along the tensile fatigue direction (0°/90°), mostly a crack in wefts (transverse yarns) appears causing an initial significant loss of stiffness. In turn, the 45° oriented laminated composite with respect to the acting force does not have the transverse yearns. In consequence, the loading force is transferred through all components. Previous studies on the laminated composite with woven fabric of the same orientation [5] demonstrated, that during the cyclic loading the cavities appeared in the pocket resin corners and a damage mode developed during fatigue aging in these regions is directly dependent on the quality of the fiber/matrix interface.

The main aim of this research was to provide a wider knowledge regarding the glass woven fabric reinforced by thermoplastic resin laminates subjected to low and high uniaxial cyclic loading, and subsequently to assess an influence of the loading history on the impact resistance at low values of velocity. Previous studies on the new Elium acrylic based laminated composites were mainly focused on studies devoted to the mechanical properties, the impact resistance for the resin modified by the rubber based copolymers. In our paper the just mentioned, properties were determined for the resin reinforced by the glass fibers. To the best our knowledge, no results are available from tests of this material carried out in order to describe the impact resistance after fatigue ageing.

Two GFRE of four ply laminates were taken into account. The first one was with a woven fabric orientation of [0°/90°]_4_, and the second one was of [45°/45°]_4_. In the preliminary tests of the experimental program, the stress–strain tensile curves were determined for both types of specimens ([0°/90°]_4_ and [45°/45°]_4_). Subsequently, two stress levels were chosen in each group for the planned impact tests. A response of the materials on cyclic fatigue loading was evaluated in terms of the stiffness variation analysis. Therefore, the elastic modulus was measured using an extensometer during tensile tests carried out between subsequent blocks of the fatigue loading cycles. The fatigue aging program was interrupted prior to the specimen failure at the given value of the stiffness drop. Subsequently, the aged specimens were dismounted from the testing machine and subjected to low-velocity impact tests using a drop weight tower. Variations of the impact energy and fibers orientation of the aged and as-received specimens were studied. Damage characterization was executed by means of the micro-tomography in order to identify the mutual relationship between the impact resistance and type of damage.

## 2. Materials and Methods

### 2.1. Material and Specimens

The material tested was a glass fibers based thermoplastic Elium acrylic. Elium 150 acrylic resin was developed and provided by the chemical group ARKEMA (Lack, France). It exhibits similar mechanical properties to that of Poly(Methyl Methacrylate) (PMMA). The glass transition (T_g_) temperature of Elium 150 is around 105 °C. In addition to the acrylic monomer (Elium), it also contains ~2.5% of the peroxide compounds (Luperox^®^) that causes a polymerization reaction at ambient temperature. A bidirectional woven glass fabric (plain weave) provided by Chomarat Textiles Industries (Le Cheylard, France) were used as the reinforcement. The material consisted of fibers intersecting themselves in the warp and weft directions. According to the datasheet, the fabric has the same properties along both these directions. The repetition period of the fabric pattern is T = 7.8 mm and its fabric mass area (surface density) is close to d_s_ = 600 g/m^2^.

The vacuum infusion method was used to the plates production, as more broadly described in [9]. This process involves the vacuum injection of a low viscosity resin into a laminate (woven). The layers of reinforcements are placed on a dry glass plate. The set is covered by a vacuum bag, which must be properly sealed. The assembly is then placed under vacuum. The vacuum allows the compaction of the laminate, while ensuring that there are no air leaks. The accelerated resin in which the peroxide catalyst (Luperox) was added, pre-mixed by blending, is introduced by means of a pot (reservoir) and a pipe (resin flow channel). The vacuum established by a vacuum pump enables the resin to migrate through the laminate until the upper woven is completely impregnated. Prior to the composite panel preparation, the acrylic resin was stirred and degassed in order to avoid the formation of voids during the infusion process. Four layers of glass fabric woven identically oriented were stacking and impregnated with the Elium acrylic resin. In order to achieve a high mechanical performance of the laminated composite a volume fraction of the E-glass fibers was about 57.0 ± 0.6% for specimens of [45°/45°]_4_ orientation and higher than 59.0 ± 0.8% for [0°/90°]_4_ orientation. These values were verified by Thermogravimetric Analysis (TGA) using Q5000IR model (TA Instrument, United States).

**Figure 1:**
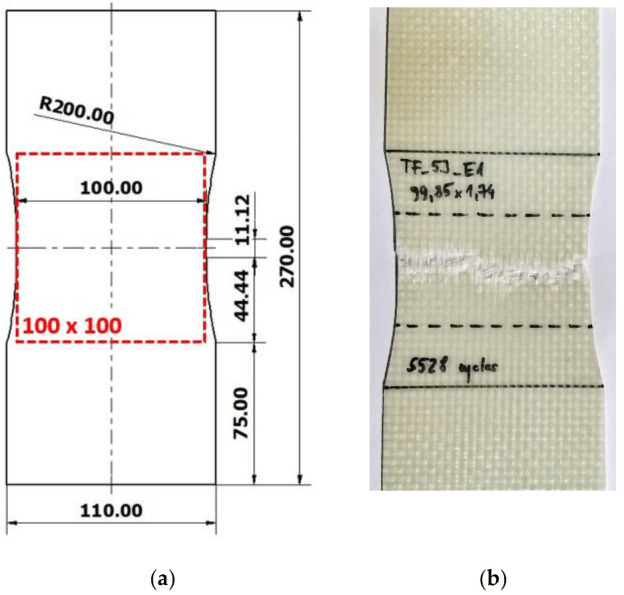
(**a**) Fatigue aging specimen with the shape adapted for the low velocity impact test; (**b**) damage localization in GFRE [0°/90°]_4_ specimen subjected to 5.5 × 10^3^ low cyclic fatigue (LCF) loading.

A nominal thickness of the manufactured specimens was about 1.75 mm. The shape and dimensions of the specimens used for fatigue aging tests are presented in Figure 1a. The size of the measuring part (dashed red line) was 100 × 100 mm and corresponded to the size of the specimens subsequently used during the impact tests. The shape and dimensions of the specimens were designed to ensure a good homogeneity and concentration of the stresses distribution in the impact area. It was verified through the numerous fatigue tests carried out at different values of the force applied. Figure 1b shows the location of damage resulting from fatigue loads.

It is well known that the thermomechanical behavior of the laminated composite strongly depends on an orientation of the reinforcing phase. Table 1 presents tensile properties of Elium enhanced by four layers of glass plain weave fabric for three directions of the fiber reinforcement.

As the composite containing the woven plain weave had the identical mechanical properties in the warp [0°] and weft [90°] (in the plane) directions, only two orientations, representing two different cases were selected in this study. The first group (rigid) was cut out along the fibers direction, while the second one (ductile) along direction inclined by an angle of 45° with regard to that of the fibers one, named GFRE [0°/90°]_4_ and GFRE [45°/45°]_4_, i.e., the strongest and weakest toughness of the material tested, respectively.

### 2.2. Fatigue Aging

**Figure 2:**
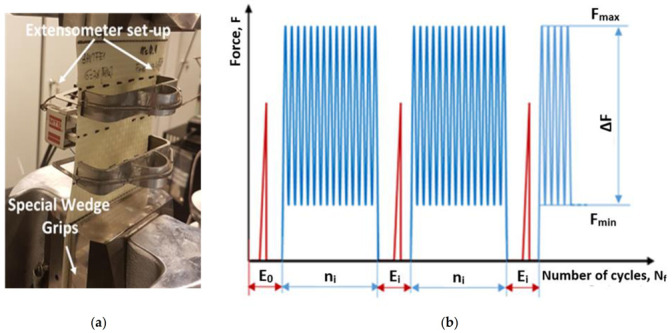
(**a**) Experimental setup for fatigue aging tests; (**b**) schematic illustration of the loading sequences.

The MTS 810 uniaxial servo-hydraulic testing machine (MTS Systems Corporation, Eden Prairie, MN, United States) of the loading capacity up to 250 kN was used in all fatigue experiments. A special gripping system was elaborated, Figure 2a, that enabled to fix specimens in the jaws of the testing machine. In order to determine a stiffness variations during tests a MTS strain extensometer of the range within ±0.2 was applied. 

Cyclic loading was carried out under force control and frequency of 2 Hz. The sinusoidal loading mode was characterized by R = F_min_/F_max_ = 0.1.

A fatigue program was divided into two types of blocks distinguished by blue and red colors, Figure 2b. In the first type blocks, the elastic modulus was determined based on a simple tensile test. A maximum force during each tensile test was lower than that corresponding to the mean loading level during fatigue aging process executed in the second block. In the second type of loading blocks, a given number of uniaxial tensile cyclic loading n_i_ was applied. The recorded elastic modulus evolution was used either to identify the pre-critical state or to interrupt the cyclic loading process prior to total failure of the specimen.

**Figure 3:**
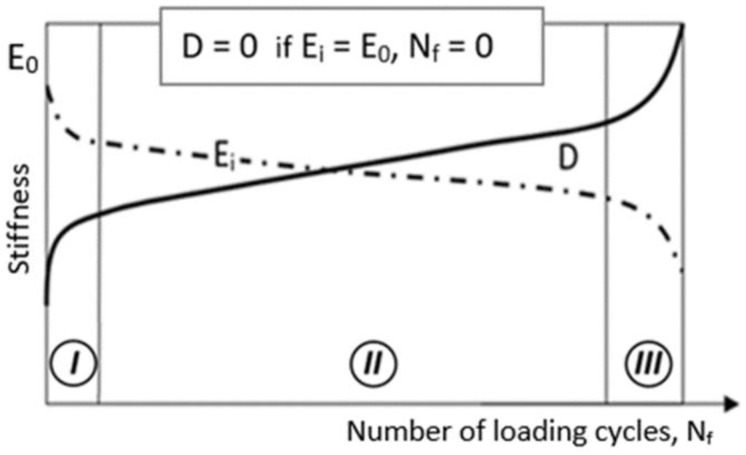
Illustrative diagram of an evolution of damage during uniaxial tensile cyclic loading.

Based on the observation of the elastic modulus evolution recorded during all fatigue conditions, a damage parameter D was defined, as seen in Figure 3. By analogy to the damage definition introduced by Kachanov [16] or Azouaoui, et al. [17], the loss of material stiffness as a damage indicator was applied. In such a case damage can be defined by a scalar parameter D in the following way:

D(N_f_) = 1 − E_i_(N_f_)/E_0_, (1)

where E_0_ is the initial stiffness (undamaged material—as received), E_i_ is the residual stiffness of aged material for a given number of cycles n_i_, N_f_ = $\sum_{i=1}^{k}n_{i}$.

Based on damage parameter suggested here and accompanying loss of stiffness, the pre-critical state was determined. As a result the cyclic loading process was interrupted prior to the total failure of specimen at the assumed damage state. It has to be mentioned here, that all the aged specimens acquired the same level of degradation (similar stiffness loss). The aged specimens were subsequently subjected to low impact velocity tests on the drop weight tower.

### 2.3. Low Impact Velocity Tests

**Figure 4:**
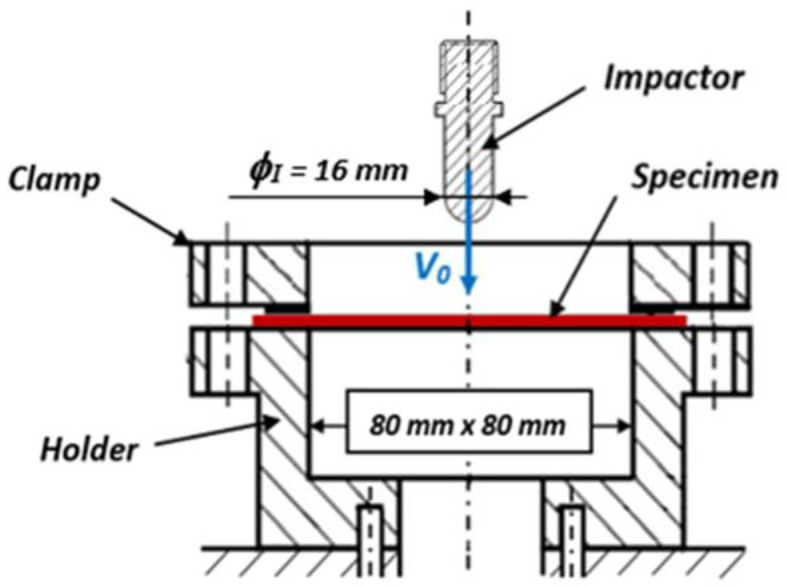
Experimental setup used for the low impact velocity tests.

The low impact velocity tests were performed on an Instron DYNATUP 9250HV drop weight tower. This testing stand, equipped with a thermal enclosure, enabled us to perform tests at a room temperature of about 20 °C. The impactor used for the impact tests had a hemispherical shape with a diameter of 16 mm. The total mass, including carriage and impactor, was equal to 5762 ± 100 g. The square shape specimens of 100 mm × 100 mm × 2 mm dimensions were fixed in a testing stand by the special clamping system, as shown in Figure 4. The griping system for impact tests at low velocity consisted of a special holder and clamp to fixed properly each tested specimen. The drop-weight tower device was equipped with a special transducer that contained the strain gauge/piezoelectric and infrared sensor. Its position was adjustable by means of the measuring system. A displacement was measured by this sensor with an accuracy of ±0.2 mm. In order to avoid some oscillations or signal noise induced by the voltage signal output from the force sensor, the fast Fourier transformations were applied for data filtration and subsequent analysis. The higher frequency components were filtered, and therefore, only the harmonics of the basic frequency responses could be seen, as reported in [18,19].

In the first step of experimental program for impact testing investigations were carried out on the as-received specimens. They were cut out to ensure the same orientations as those for fatigue tests. Impact tests were carried out at room temperature for the impact energies of 5, 10, 30 and 50 J, which corresponded to the impact velocity equal to 1.32, 1.86, 3.23 and 4.16 m/s, respectively. The same values of energy were used for impact testing of the prior aged specimens (see Table 2). The tests were repeated five times for each value of impact energy in order to check a repeatability of the results. The force and displacement were registered during each test.

Based on the force and displacement variations of the impactor, the energy changes were calculated. Such changes represent the energy transfer from the projectile to the composite specimen. The energy change history for each impact test was determined by proper integrating the velocity change plots, i.e., the area under the curve representing these changes. In the test, the absorbed energy was determined as a fraction of the energy transferred from the projectile to the composite specimen at the end of the impact, and in each case the impact energy corresponded to the kinetic energy of the projectile just before the impact. Although the maximum force, contact duration, history of force and displacement changes and energy are important characteristics of composite laminates subjected to dynamic loads, only the change in the amount of energy absorbed was the main parameter considered in the experiment. For this reason, studies were carried out on the influence of fatigue load on impact toughness, and especially its negative effect in the form of an increase in energy dissipation due to the loss of stiffness. Impact resistance was quantified on the basis of the analysis of the maximum impact force F_i_ and the corresponding displacement U, as well as the absorbed energy E_a_ and the penetration threshold. In order to visualize the type and extent of damage, microstructural tests were carried out with the use of SEM (JOEL Ltd., Akishima, Japan) and CT scan (RX SOLUTIONS, Chavanod, France) for aged and non-aged specimens subjected to the impact test. Microtomographic observations were carried out on the prismatic specimens with dimensions of 50 mm × 50 mm × 2 mm, with the power of the X-ray beam of 80 kV and spatial resolution of 1.0 μm.

## 3. Results and Discussion

### 3.1. Residual Stiffness Examination

The reliable analysis of the stiffness evolution for the polymer based woven fabric composites under cyclic loading requires fatigue damage evaluation in the area of measurements. Therefore, a specific shape of the specimen gauge length was applied. It enforced the highest stress concentration in its smallest section. Such a design also enabled a local monitoring of the elastic modulus evolution by means of the extensometer. This possibility is particularly important due to the fact that the generation of damage resulting from local plastic deformation in the areas of stress concentration (around voids and other structural imperfections) tends to homogenization in the selected cross-sections. Thus, in this area, matrix cracks initiate and propagate in the transverse yarns. In order to investigate these phenomena, the MTS model 634.31F-24 longitudinal extensometer was used in the tests, adapted to the specimen gauge length of dimensions from 10.0 to 20.0 mm with the possibility of step adjustment +4.0/−2.0 mm. According to the manufacturer, this device ensures high accuracy of measurements in accordance with the ISO 9513 class 0.5 standard. The maximum error in the measuring range is about 0.21%. 

**Figure 5:**
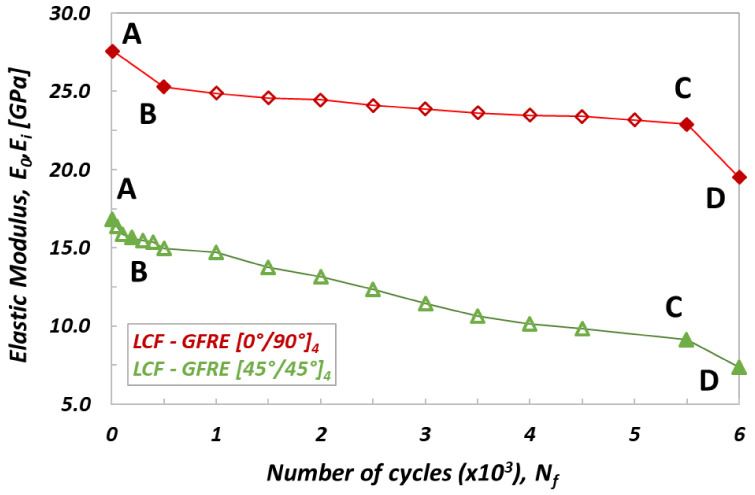
Variation of the elastic modulus versus number of cycle for specimens of different fibers orientation obtained for LCF tests. AB—crack initiation (crack in the transverse yarn), BC—crack growth (interlaminar debonding), CD—ultimate failure (fibers breakage).

**Figure 6:**
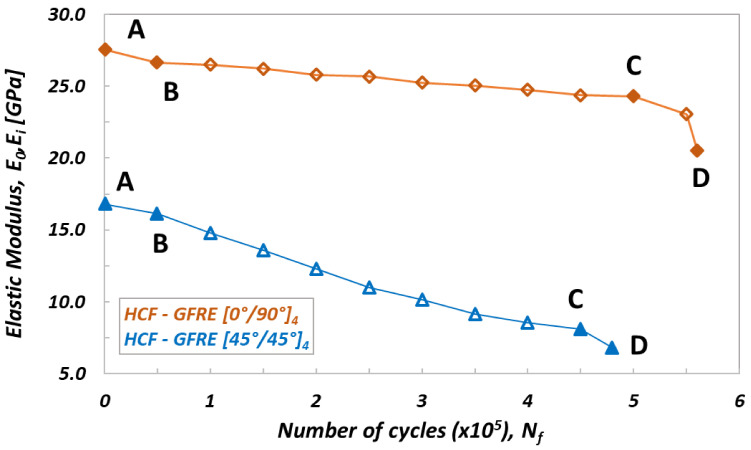
Variation of the elastic modulus versus number of cycle for specimens of different fibers orientation obtained for HCF tests. AB—crack initiation (crack in the transverse yarn), BC—crack growth (interlaminar debonding), CD—ultimate failure (fibers breakage).

All fatigue tests started by the Young’s modulus (E_0_) determination, and subsequently the selected blocks of loading cycles n_i_ were executed. After the last cycle the specimen was unloaded to zero force and the residual elastic modulus E_i_ was determined. Variations of the elastic modulus determined under cyclic loading for LCF and HCF tests are presented in Figure 5 and Figure 6, respectively.

The stress levels in the LCF tests were higher than the yield stress. For specimens oriented as [0°/90°]_4_ the stress level was equal to 250 MPa (i.e., 35 MPa above the yield stress—see Table 2). In the case of specimens oriented as ([45°/45°]_4_) it was 80 MPa (i.e., 22 MPa above the yield stress, Table 2). Each block employed for reduction of the stiffness of tested material contained n_i_ = 500 loading cycles. As observed in Figure 5, the variation of stiffness with a number of cycles exhibits three clearly marked zones, for both materials tested. In the first part of the curves (up to point B), its value drops rapidly, as the result of typical fatigue behavior of composite materials, that leads to some stiffness loss (damage in the early fatigue, mainly develops in the matrix) [13,14]. In the second part (BC zone in Figure 5) a degradation of the matrix and delamination process are further developing, however much slower than during the first stage of fatigue. The elastic modulus decreases almost linearly, however, for GFRE [45°/45°]_4_ more than for the GFRE [0°/90°]_4_. In the last part of the process, beyond point C, there is a relatively abrupt drop of the elastic modulus leading as a consequence to the fibers breakage, which takes below point D.

A similar program was arranged for HCF tests, however, for the stress levels higher than the yield stress. The maximum stress levels were equal to 80 and 55 MPa for [0°/90°]_4_ and [45°/45°]_4_ composite laminates orientations, respectively (Figure 6). In this case, the total number of cycles to failure N_f_ was about 500.000 for both orientations considered. Each of the loading blocks applied in the experimental program comprised n_i_ = 50.000 cycles.

Similarly to the characteristics presented in Figure 5, also on curves describing an evolution of residual stiffness for HCF tests (Figure 6), three zones can be distinguished: AB, BC and CD. One can noticed however, that during the HCF tests the initial stiffness decreases a bit slower (zone AB) than that observed for the LCF tests, and as consequence, the curves pass smoothly to the second stage (BC zone) where the process of cyclic softening for both woven fabric orientations is practically the same as that for the specimens subjected to LCF. Finally, beyond the point C a similar character of the curves variations can be observed for both orientations taken into account. As it is shown in Figure 5 and Figure 6, almost 17% reduction of the initial stiffness was obtained for the GFRE [0°/90°]_4_ independently, whether the LCF or HCF tests were executed. In the case of GFRE [45°/45°]_4_, the reduction of stiffness was much clearer and amounted to almost 48%.

### 3.2. Identification of the Pre-Critical State

**Figure 7:**
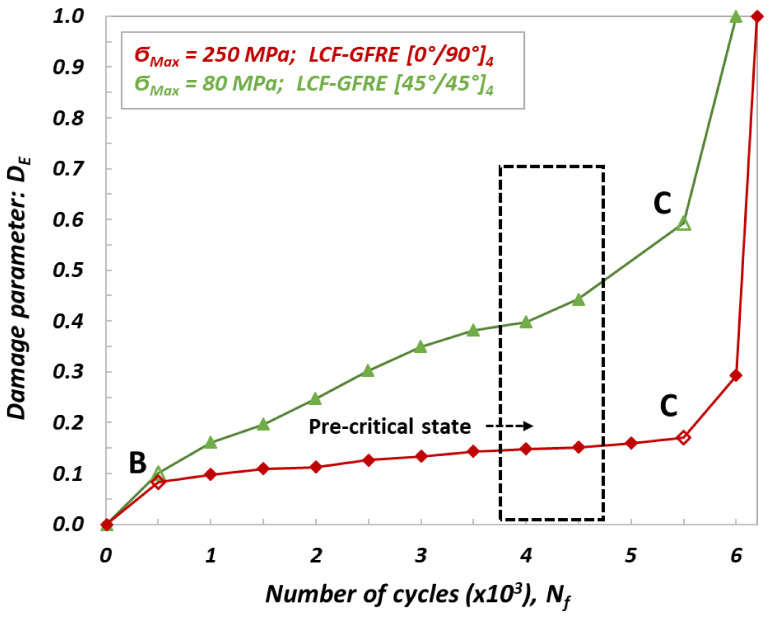
Evolution of damage parameter D_E_ determined from LCF tests for GFRE [0°/90°]_4_ and GFRE [45°/45°]_4_.

**Figure 8:**
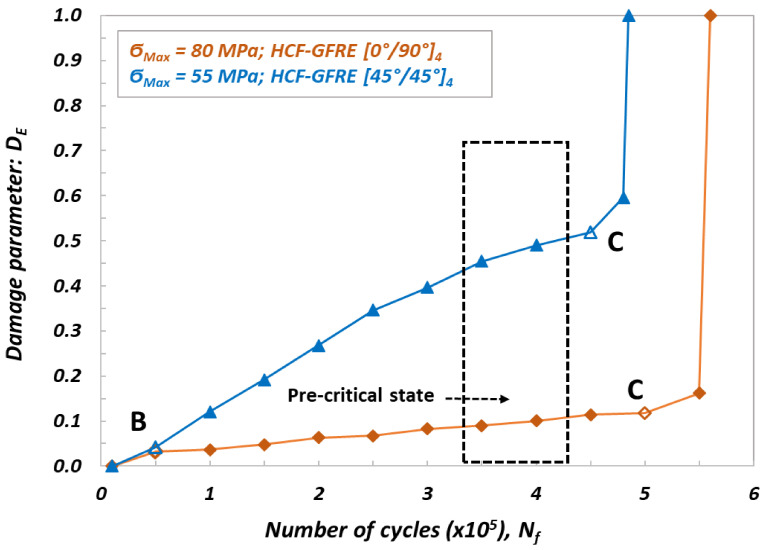
Evolution of damage parameter D_E_ determined from HCF tests loading for GFRE [0°/90°]_4_ and GFRE [45°/45°]_4_.

As observed, the cyclic loading strongly affected the stiffness of the materials tested. Depending on the woven fabric orientation and number of cycles to failure, a minor or major decrease in stiffness was identified. The results are consistent with those presented in previous studies on fatigue behavior of glass plain-weave fabric composites in on- and off-axis directions, although in this study the Elium thermoplastic resin was used as a tackifier instead of thermoset epoxy [15,20]. In order to determine a pre-critical state (i.e., the value of the maximum internal damage, at which the sample will not be destroyed), the damage parameter D_E_ was calculated. It was specified based on the value of the measured residual stiffness E_i_ for a given number of cycles N_f_, by using equation 1. Its values for the specimens subjected to LCF and HCF loading are presented in Figure 7 and Figure 8, respectively.

Taking into account evolutions of the damage parameter presented in Figure 7 and Figure 8, one can conclude that three stages of damage can be easily noticed. In the first stage represented by a part of characteristic denoted as 0 B, the damage parameter exhibits a significant increase and takes the values at the end of this stage of D_E_ = 0.085 ÷ 0.100 and D_E_ = 0.035 ÷ 0.040 for LCF and HCF tests, respectively. In the second stage, defects in the form of micro cracks and delamination, generated by the fatigue aging, were increasing much faster for GFRE [0°/90°]_4_ oriented laminate composites than that for GFRE [45°/45°]_4_ ones. For the GFRE [45°/45°]_4_ composite laminates, D_E_ parameter increases with the number of cycles to failure, only up to 0.15 and 0.10 for the LCF and HCF tests, respectively. In the case of GFRE [0°/90°]_4_ the same parameter at the same stage of damage takes the value equal to 0.6 independently of a loading type (LCF or HCF). The third stage represents a very short final phase that covers a time to reach the failure. It appeared at N_f_ = 500.000 for the HCF and at N_f_ = 6.000 for the LCF.

Based on the residual stiffness E_i_, and damage parameter D_E_ evolutions, the pre-critical states were established for all groups of the composite laminates tested. Their ranges are marked by the dotted black lines shown on Figure 7 and Figure 8. Identification of the pre-critical states enabled to interrupt the processes of aging, induced by uniaxial cyclic loading at a given value of the stiffness loss, and to maintain a comparable character of the internal damage (micro cracks in matrix and interlaminar debonding) for both orientations taken into account and stress levels applied. Therefore, the specimens for impact tests made of the GFRE [45°/45°]_4_ were aged until damage parameter D_E_ attained the value of 0.45, that corresponded to the 𝜎_max_ = 80 MPa and N_f_ = 4.500 cycles for LCF tests. For the same orientation specimens subjected to HCF under 𝜎_max_ = 55 MPa and the same value of D_E_ = 0.45, 350.000 cycles were necessary to attain the pre-critical phase. Regarding the material with woven fabric oriented along the force direction (GFRE [0°/90°]_4_), the maximum safe value of the damage parameters was equal to D_E_ = 0.15 and D_E_ = 0.11, for 𝜎_max_ = 250 MPa at LCF tests and 𝜎_max_ = 80 MPa at HCF test, respectively. Hence, to reach the assumed value of stiffness loss the N_f_ = 4.000 cycles for LCF and N_f_ = 400.000 cycles for HCF were required. Finally, in order to study the effect of softening generated by the fatigue aging on the impact resistance, 80 specimens for impact tests of 100 mm × 100 mm sizes were aged by uniaxial cyclic loading, 20 for each tested group.

### 3.3. Effect of Fatigue Aging on Impact Properties

Four groups of prior aged specimens were impacted using a drop tower with energy levels of 5 J, 10 J, 30 J and 50 J. The stiffness loss introduced by fatigue was approximately equal to 12% in the group of specimens cut out along the yarns (LCF and HCF tests on GRFE [0°/90°]_4_), and 40% for specimens with woven fabric inclined at 45° with respect to the acting force (LCF and HCF test for on GRFE [45°/45°]_4_). In order to study an effect of aging on the impact properties, the results were compared with those carried out on the non-aged specimens with the same woven fabric orientations. The low velocity impact test results are reported in Table 3 and Table 4.

**Figure 9:**
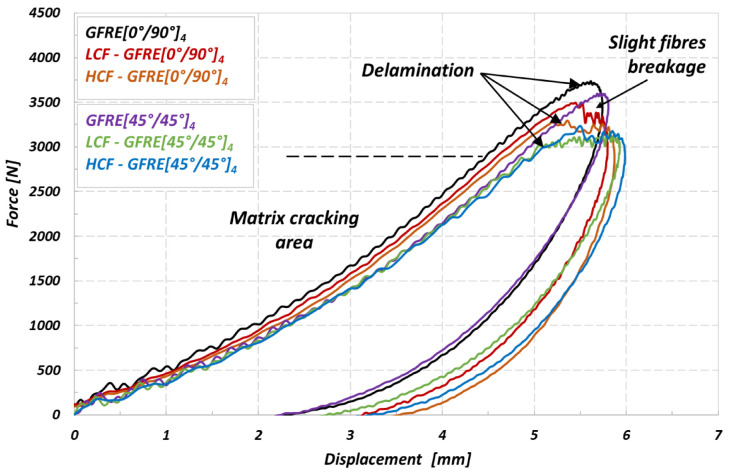
Force–displacement curves of the non-aged and aged GFRE [0°/90°]_4_ and GFRE [45°/45°]_4_ due to the LCF and HCF ageing processes, the results are for an impact energy of 10 J.

The most common way to present the low velocity impact response and resulted damage is to use the recorded impact histories of force-time or force-displacement. Before an analysis let us to define some important quantities like a peak force and adsorbed energy. Peak force corresponds to the maximum value of impact force (F_i_) registered during the contact between the impactor head and specimen. Absorbed energy should be treated as the amount of energy transferred from the impactor to specimen at the end of test. The impact force history at the low impacts velocity provides important knowledge regarding a damage initiation and its further propagation [21], and therefore, a particular emphasis should be taken into account with regard to signal acquisition quality. Hence, the oscillations of higher frequency values in recorded data of force signals were filtered using the FFT techniques. As consequence, only harmonics of responses for basic frequency level are presented on the curves. Figure 9 presents the force versus displacement curves for composite laminates subjected to an impact energy of 10 J. The early stages of the force versus displacement curves (slope of the curve) are not the same in case of the GFRE [0°/90°]_4_, indicating that fatigue aging process affected the laminate’s stiffness. Subsequently, some small oscillations can be observed with force increase exhibiting a presence of matrix cracking [22]. The effect is mostly visible for GFRE [45°/45°]_4_, as a contribution of resin in the force transmission is more significant for this kind of composite. It takes place for a force value of about 2000 N. Above 3000 N an effect of the force stagnation can be observed. It was due to the reduction of bending stiffness resulting from the brittle impact damage behavior of GFRE, and moreover, from the delamination process development. The intensity of the force stagnation process was clearer for the unharmed (non-aged) specimens than that of the aged composite laminates. A decrease of the maximum impact force (F_i_) was obtained for both orientations of the reinforcement considered in comparison to the non-aged composite. It was associated with an increase of the deflection, identifying material ability to dissipate more energy.

**Figure 10:**
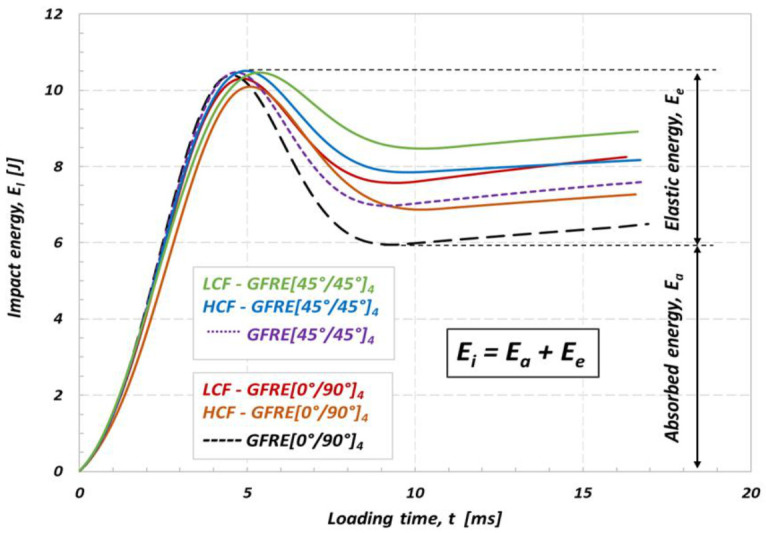
Effect of fatigue aging on the absorbed energy of the non-aged and aged GFRE for impact energy equal to 10 J at 20 °C.

In order to better understand the effect of uniaxial fatigue aging on the low impact velocity properties and damage appearance in the material tested, the curves representing an energy variation versus time were elaborated and plotted in Figure 10. The impact resistance represents an ability of the material to absorb energy without depicting too many other obvious damage indicators. The impact energy which corresponds to the peak energy on the E_i_(t) diagram can be decomposed into two parts, the absorbed energy which generates damage, and elastic energy that serves for the impactor rebound. Therefore, in the present work, the energy absorbed by the specimen (E_a_) is used as an indicator of damage degree [19,23]. Impact energy equal to 10 J, was insufficient to cause a complete loss of strength and penetration even for the aged specimens. However, it has to be noticed, that the composites subjected to prior LCF loading exhibit much lower energy dissipation ability. The results in Figure 9 enable to assess a significant loss of stiffness caused by matrix cracking and interface debonding between the fibers and the matrix. The results for GFRE [45°/45°]_4_ subjected to low velocity impact at 10 J exhibit that it was a less prone to delamination than GFRE [0°/90°]_4_ and kept better impact resistance after fatigue aging. The values of absorbed energy by the composite laminates are reported for different aging processes, in Table 3.

**Figure 11:**
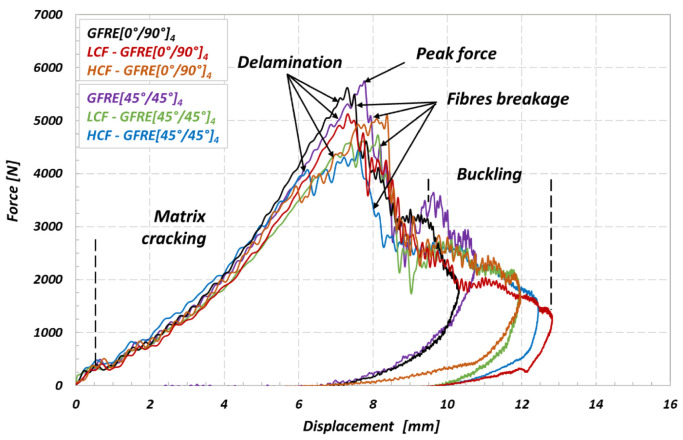
Force–displacement curves of the non-aged and aged GFRE [0°/90°]_4_ and GFRE [45°/45°]_4_ under different aging processes, the results are for impact energy of 30 J.

**Figure 12:**
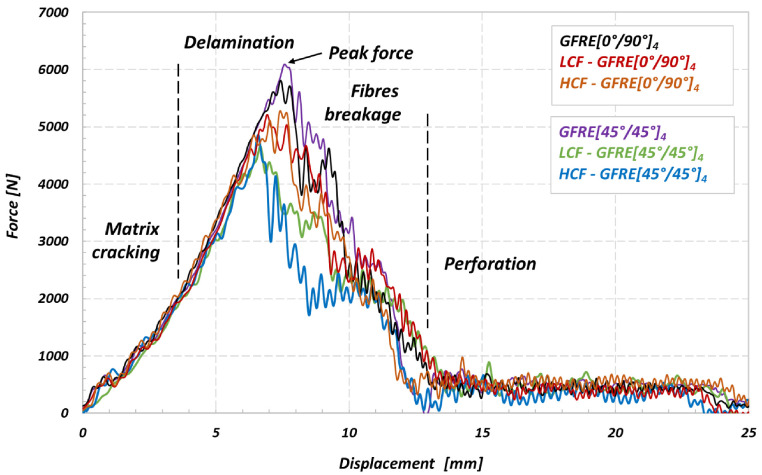
Force–displacement curves of the non-aged and aged GFRE [0°/90°]_4_ and GFRE [0°/90°]_4_ under different aging processes, the results are for impact energy of 50 J.

Subsequently, all groups of materials tested were subjected to the impact tests at 30 and 50 J. Figure 11 and Figure 12 show the evolution of the force versus displacement of the materials. All curves were obtained at room temperature (20 °C).

It is clearly visible in Figure 11 that an initial slope of the characteristics slightly decreased due to prior in plane uniaxial cyclic loading applied. Such an effect proves that the internal damage caused a stiffness reduction in out of plane direction due to matrix cracking and softening of the reinforcing phase, and as a consequence, led to partial loss of the impact dissipation ability.

The impact energy values of 30 and 50 J were sufficient to cause a visible fracture with numerous internal cracks. Both aged and non-aged specimens did not resist the impact under 50 J, and all of them were perforated. It should be noted that again the aged specimens representing GFRE [45°/45°]_4_ orientation were the least resistant to impacts. In this case, the cracks of the matrix occurred under the force lower than that for GFRE [0°/90°]_4_. Moreover, a delamination appearing during impact led to the significant reductions of the transmitted force. Hence, the peak force oscillations recorded for these specimens reached even 1000 and 1200 N approximately, for the impact tests at 30 and 50 J, respectively.

**Figure 13:**
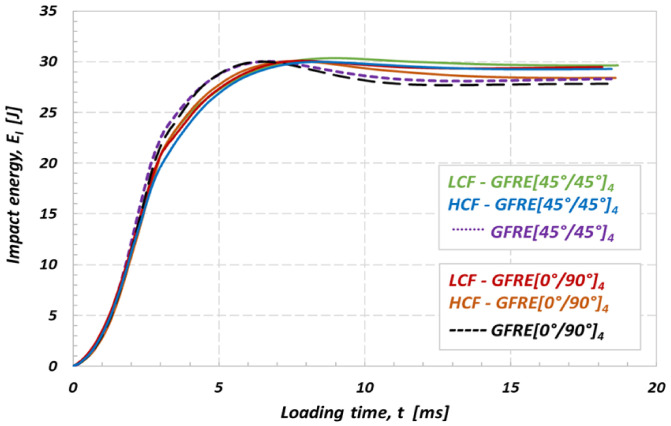
Effect of fatigue aging on the absorbed energy of the non-aged and aged GFRE for impact energy of 30 J at 20 °C.

**Figure 14:**
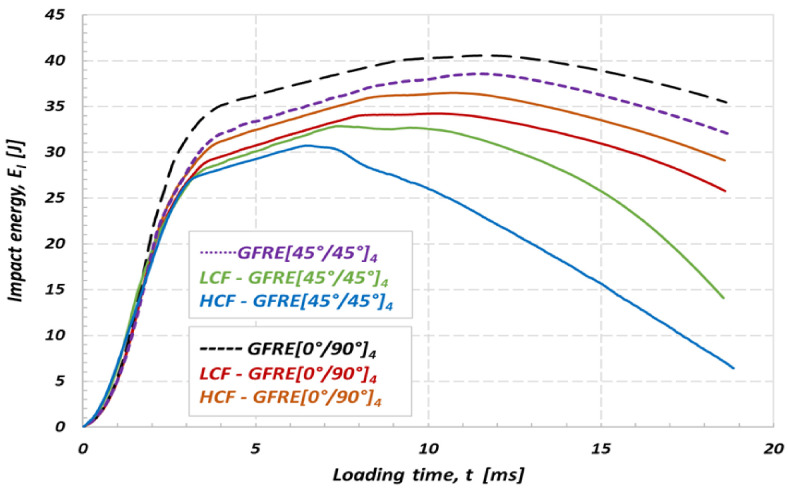
Effect of fatigue aging on the absorbed energy of the non-aged and aged GFRE for impact energy of 50 J at 20 °C.

The energy diagrams shown in Figure 13 and Figure 14 exhibit that values of energy absorbed by GFRE [45°/45°]_4_ subjected to prior LCF or HCF loading are considerably lower if compared to the other ones obtained in this research. Therefore, the GFRE [45°/45°]_4_ ability to dissipate energy is practically negligible low. One can indicate such an effect looking on the course of the green line for example, that almost does not drop. This fact evidences that the energy of the impactor is equal to the absorbed one, approximately. In consequence, the GFRE [45°/45°]_4_ under LCF loading (represented by the green line) in Figure 13 was more damaged during the impact loading expressed by a relatively large delamination region.

A comparison of the results presented in Figure 13 and Figure 14 enabled to conclude that impact energy of 30 J may be treated as the amount of energy close to the impact strength limit for composites tested. All energy characteristics exhibit energy peak, and further, their courses start to going down that is a clear evidence of the penetration and subsequent perforation of the specimens tested. Moreover, the differences in courses of the impact energy observed for the as-received and fatigue aged material directly identify an effect of fatigue aging on its impact resistance. This is an additional fact confirming previous observations, that the aged GFRE [45°/45°]_4_ material is the weakest one among all considered in this research. 

Having the characteristics presented in Figure 14 it is easy to identify a rupture initiation in specimens tested. In the case of non-aged material the rupture started to develop when the energy level attained 40 J and 38 J, approximately, for the GFRE [0°/90°]_4_ and GFRE [45°/45°]_4_, respectively. Furthermore, it is clearly visible that all the groups of specimens after fatigue loading exhibited a significantly lower impact resistance. In the most undesirable case (HCF-GFRE [45°/45°]_4_) the perforation appeared.

### 3.4. Identification of the Penetration Threshold

In order to assess a real improvement of the impact resistance of Elium acrylic based composite, the penetration threshold curves were fitted for the three materials. In fact, it is well known that the penetration threshold belongs to the most important features, enabling better classification of the impact properties of the laminated composites [22]. This parameter determines the energy required for perforation of the laminated composite. In the present study a method defined by Reis et al. [24] and Aktas et al. [25] was applied. The authors defined an energy profile diagram (EPD) that is useful to compare the impact and absorbed energies, as well as to identify the penetration and perforation thresholds. According to Aktas et al. [25], the penetration threshold can be defined as the point where the absorbed (E_a_) and impact (E_i_) energy are equal.

**Figure 15:**
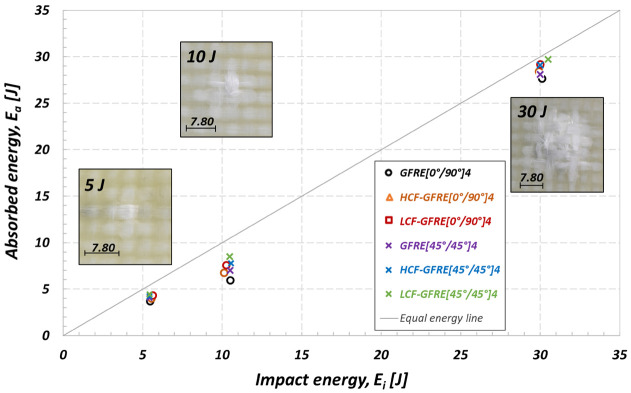
EPD used to analysis of the effect of fatigue aging on the penetration threshold of the GFRE at 20 °C.

Figure 15 shows the EPD for all groups of specimens at ambient temperature. The diagram presented in Figure 15 summarizes the results obtained for the as-received and aged material subjected to impact under energy levels equal to 5, 10 and 30 J. Data points of both unharmed and aged laminate are located below the line representing equilibrium between the applied and dissipated energy. It means the penetration threshold was not achieved, and as a consequence, the applied energy is used to bounce of the impactor. For the highest impact energy considered (50 J) all specimens tested were perforated.

The results for the impact energy of 10 and 30 J clearly show how the difference in energy absorption ability may change due to a type of reinforcement orientation and fatigue aging conditions. The prior aged laminates under LCF loading conditions attained practically the impact resistance limit regardless of the woven fabric orientation. It means that the absorbed energy attained the maximum possible amount of the impact energy applied. The highest values of absorbed energy were obtained for the GFRE [45°/45°]_4_ after ageing due to LCF tests. It means that—in comparison to the other considered material configurations—such an oriented material is the least suitable for applications where the impact loading is dominant. Contrary to that case, the lowest values of absorbed energy were achieved after ageing due to HCF tests for GFRE [0°/90°]_4_. In order to illustrate damage occurred on the opposite side of the impacted specimens, damage images were included in Figure 15. They represent stages of damage for the GFRE [0°/90°]_4_ in the as-received state subjected to impact under energy equal to 5, 10 and 30 J.

**Figure 16:**
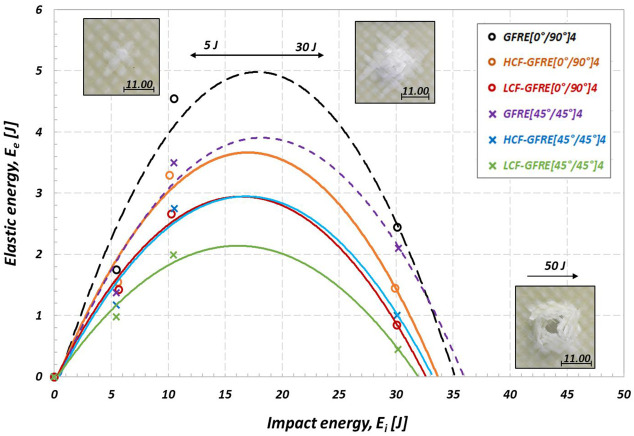
Elastic energy versus impact energy for assessment of the effect of fatigue aging on the penetration threshold of the GFRE at 20 °C.

The authors also defined another approach in which a diagram of the elastic energy (E_e_) versus impact energy (E_i_) is used. The elastic energy is calculated as a difference between the absorbed impact energy and that corresponding to the peak of force (incident impact energy). The roots of the corresponding second degree polynomial equations fitting experimental data give energy values where impact energy (E_i_) is equal to the absorbed energy (E_a_), i.e., where E_e_ = 0. The roots of higher values indicate the penetration thresholds for laminates [9,24]. The values of the penetration thresholds for the non-aged and aged GFRE are shown in Figure 16.

One can observe that the penetration thresholds calculated at 20 °C for impact energies of 5, 10 and 30 J strongly depend on the internal damage introduced by fatigue aging. As expected, the non-aged composites represented materials of the best impact resistance properties. An implied value of the impact energy at penetration thresholds was equal to 35.8 J for the GFRE [45°/45°]_4_ and 35.1 J for GFRE [0°/90°]_4_. In the case of the same materials after ageing due to HCF tests the impact energy at penetration thresholds was equal to 33.2 and 33.5 J, respectively, and for the same materials after ageing due to LCF: 31.9 and 32.6 J, respectively. It is easy to notice that, the maximum difference between penetration thresholds determined is only 3.9 J. The main reason for such a small difference results directly from the limited number of data points (only 3 energy levels were used in the polynomial fitting).

Despite the limited number of data available, one can conclude that the history of the elastic energy variation due to increasing impact energy provides an effective parameters for analysis of the impact response of composites reinforced by glass fibers of different woven fabric orientations. Thanks to the ΔE_e_ a difference between the elastic energy of the non-aged and aged composite can be easily determined. It can be observed that the difference between the elastic energy for the non-aged and aged GFRE [45°/45°]_4_ (ΔE_e_) increases with the increase of the impact energy applied. An opposite effect takes place in the case of GFRE [0°/90°]_4_, particularly, if tests were carried out at energy values close to that corresponding to the perforation limit.

The results enable to conclude that the fatigue ageing process decreases the elastic response expressed by the stiffness reduction of both composites tested, and as a consequence, affects their toughness. It is strongly dependent on the mechanisms developing during tension on one hand, and a cohesion forces reduction between fibers and resin due to the fatigue ageing on the other. It leads to cracks generation in the transversal yarns for GFRE [0°/90°]_4_, and either in warps or wefts for GFRE [45°/45°]_4_.

### 3.5. Damage Analysis of Impacted Composite Laminates

Figure 15 and Figure 16 contain photos of the reverse sides of the selected impacted plates for both fiber orientations tested at different energy values. At low impact energy, the damage is localized mainly in the matrix and takes the form of numerous cracks. For tests carried out at higher values of the impact energy, the area of damage increased significantly, revealing an occurrence of more severe forms of destruction such as delamination and fibers breakage, for example. When the composite laminates were subjected to prior fatigue aging, a degree of damage generated by that process underwent further development due to the impact introducing more severe forms of damage. One can indicate that the composites prestressed due to fatigue aging under the stress amplitude below the elastic limit were more damaged than those subjected to the same process, however, under stress amplitudes higher that the elastic limit. As a consequence, the mechanisms of plastic deformation were activated, which might induce some cavities and contribute to arrest propagation of cracks when the plate is subjected to impact [26,27,28]. This effect is more obvious for the GFRE [0°/90°]_4_ composite laminate.

**Figure 17:**
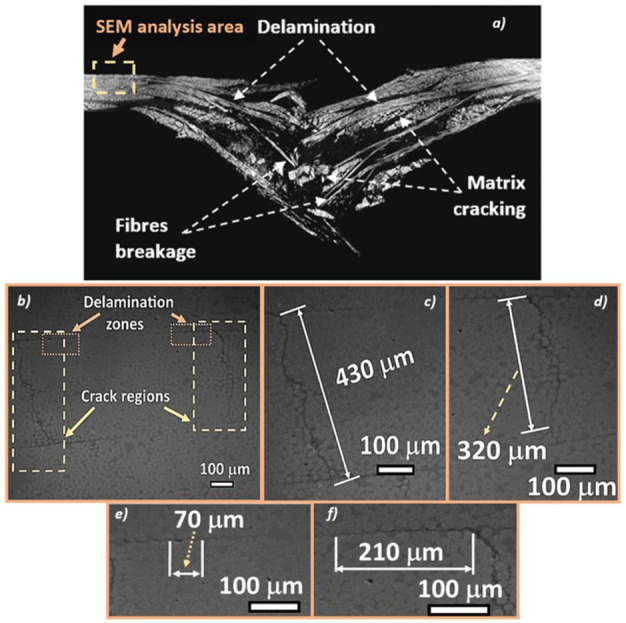
Microtomographic and SEM analysis of the aged GFRE [45°/45°]_4_, impacted at 30 J for: (**a**) general view; (**b**–**d**) intralaminar defects; (**e**,**f**) interlaminar debonding.

**Figure 18:**
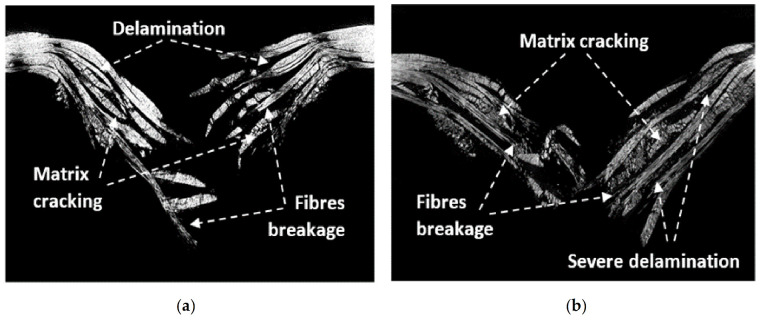
Microtomographic analysis of the impacted GFRE [0°/90°]_4_ at 50 J for: (**a**) as-received and (**b**) aged composites.

The difference in damage that exists between the [0°/90°]_4_ and [45°/45°]_4_ orientations is that the two systems do not have the same glass fibers weight fractions. These observations are confirmed by the tomographic analyses performed on the specimens of [45°/45°]_4_ and [0°/90°]_4_ orientations, which were subjected to fatigue aging (Figure 17 and Figure 18). An appearance of the fatigue streaks, and the severe damage that results from them, show that the matrix cracking and delamination generated during fatigue were in fact the main causes of severely damaged areas formation.

Figure 17 shows intralaminar (Figure 17b,c,d) and interlaminar damage (Figure 17e,f) in the woven fabric composite with the warps and wefts oriented with an angle of 45 degree with respect to the force direction after fatigue loading and subsequent impact. There are clearly visible some cracks in the selected yarn (weft), as well as delamination between the neighboring layers. The cracks in the warps and wefts were induced by LCF or HCF loading leading to the softening of the whole structure of the composite. In consequence, the stiffness and impact resistance were affected. Those observations are in agreement with the SEM inspections presented in the previous study of damage induced by tensile fatigue loading in composites reinforced by glass plain-weave fabric [14].

In Figure 18, a comparison between the as-received and aged plate impacted at energy equal to 50 J is presented. The microtomography analysis showed that the area of matrix cracks of the non-aged laminate is smaller than that for the aged laminate plate observed. The results confirmed that the cyclic loading (aging) of the laminated composite leads to the decohesion of the fibers/matrix interface and promotes a generation of the severe delamination and matrix cracking when both tested composite laminates are impacted.

## 4. Conclusions

Two groups of GFRE based composite laminates, of shapes and dimensions adapted for the impact resistance testing after prior fatigue aging, were prepared by means of the infusion process. The effect of damage induced by fatigue aging was investigated through analysis of the impact resistance properties. A variation of the elastic modulus and damage morphology of both composite laminates subjected to cyclic loading were analyzed. It was observed that both tested GFRE [0°/90°]_4_ and [45°/45°]_4_ exhibited the visible effect of the stiffness loss during fatigue. The initial stiffness was reduced of about 17% for the GFRE [0°/90°]_4_, after either LCF or HCF tests. The reduction was much more pronounced in the case of GFRE [45°/45°]_4_. It was equal to 48%.

Based on the evolution of either residual stiffness or damage parameter during fatigue aging due to LCF and HCF the pre-critical states were successfully identified. It enabled determination of the damage parameter D_E_ that defines a number of cycles necessary to attain the pre-critical state for each group of the aged specimens under stress level applied. The stiffness reduction introduced by fatigue was evaluated. It was equal to 12% for specimens subjected to LCF tests and 40% for HCF ones for both composite laminates tested. 

Low impact velocity tests carried-out on the non-aged plates at different levels of impact energy exhibited their good impact resistance. Therefore, the non-aged specimens impacted at energy of 5, 10 and 30 J were not perforated and showed a great ability to rebound the impactor.

The composites subjected to LCF loading demonstrated the greatest decrease of the initial slope on the force-displacement diagrams. It was thanks to the significant reduction of stiffness caused by the matrix cracking and interface debonding taking place between the fibers and matrix. The maximum force reduction for tests carried out at the impact energy of 50 J for GFRE [0°/90°]_4_ and GFRE [45°/45°]_4_ was equal to 11% and 19%, respectively.

Analysis of the absorbed energy during impact tests, confirmed that the aged materials containing the woven fabric oriented at the angle of 45° exhibited the weakest impact resistance. Therefore, one can conclude that the fibers orientation, fatigue aging, and glass fibers concentration significantly affect the elastic properties and lead to a decrease of the stiffness reduction and increase of the energy absorbed.

The results presented in this research open many paths of enquiry relating to phenomena that can explain some particular low impact velocity behavior of the thermoplastic composites which contain a fatigue damage history. A multiscale numerical modelling of the impact resistance as well as the temperature and strain rate effects of these new materials are in process, and presumably, will provide reasonable answers to some important questions in the near future.

## Figures and Tables

**Table 1:** Mechanical properties of woven fabric for the three different orientations: φ—fibers orientation, E—Young’s modulus, σ_u_—ultimate tensile strength, σ_y_—yield stress, ε—strain to failure.

φ [°]	E (GPa)	σ_u_ (MPa)	σ_y_ (MPa)	ε (%)
0	27.5 ± 1.2	485.0 ± 19.4	215.0 ± 7.2	1.9 ± 0.1
45	16.5 ± 1.0	165.0 ± 11.2	58.0 ± 2.9	20.5 ± 1.2
90	27.5 ± 1.3	485.0 ± 18.7	215.0 ± 5.9	1.9 ± 0.1

**Table 2:** Fatigue aging conditions for both laminate composites tested under LCF and HCF.

	Specimen	𝜎_y_ (MPa)	𝜎_max_ (MPa)	F_max_ (kN)	F_min_ (kN)	R	f (Hz)
LCF	GFRE [0°/90°]_4_	215.0 ± 7.2	250.0	43.0	4.3	0.1	2.0
	GFRE [45°/45°]_4_	58.0 ± 2.8	80.0	13.7	1.3	0.1	2.0
HCF	GFRE [0°/90°]_4_	215.0 ± 7.2	80.0	14.0	1.4	0.1	2.0
	GFRE [45°/45°]_4_	58.0 ± 2.8	55.0	10.2	1.0	0.1	2.0

**Table 3:** Low impact velocity test results for all laminates at impact energy of 5 J and 10 J.

Specimen	5 J			10 J		
	E_a_ (J)	F_i_ (N)	U (mm)	E_a_ (J)	F_i_ (N)	U (mm)
GFRE [0°/90°]_4_	3.7 ± 0.1	2379.5 ± 24.7	3.85 ± 0.1	5.9 ± 0.2	3731.5 ± 72.1	5.74 ± 0.1
LCF-GFRE [0°/90°]_4_	4.2 ± 0.2	2254.2 ± 12.5	3.97 ± 0.1	7.6 ± 0.5	3494.3 ± 68.5	5.79 ± 0.2
HCF-GFRE [0°/90°]_4_	4.0 ± 0.1	2265.9 ± 36.2	3.93 ± 0.3	6.8 ± 0.4	3344.6 ± 43.9	5.87 ± 0.5
GFRE [45°/45°]_4_	4.0 ± 0.1	2229.5 ± 16.2	3.9 ± 0.1	7.0 ± 0.1	3594.5 ± 42.1	5.4 ± 0.1
LCF-GFRE [45°/45°]_4_	4.4 ± 0.2	2109.5 ± 27.6	4.2 ± 0.2	8.5 ± 0.4	3150.4 ± 54.2	5.8 ± 0.2
HCF-GFRE [45°/45°]_4_	4.3 ± 0.1	2176.7 ± 14.2	4.3 ± 0.1	7.8 ± 0.2	3235.6 ± 36.4	5.6 ± 0.2

**Table 4:** Impact test results for all composite laminates at impact energy of 30 J and 50 J.

Specimen	30 J			50 J		
	E_a_ (J)	F_i_ (N)	U (mm)	E_a_ (J)	F_i_ (N)	U (mm)
GFRE [0°/90°]_4_	27.6 ± 0.3	5623.8 ± 64.7	10.3 ± 0.1	-	5939.9 ± 90.2	-
LCF-GFRE [0°/90°]_4_	29.2 ± 0.4	5125.3 ± 112	12.8 ± 0.4	-	5212.1 ± 55.4	-
HCF-GFRE[0°/90°]_4_	28.5 ± 0.3	5116.9 ± 86.2	11.9 ± 0.2	-	5292.4 ± 116	-
GFRE [45°/45°]_4_	28.1 ± 0.3	5750.4 ± 96.4	10.7 ± 0.2	-	6019.8 ± 67.4	-
LCF-GFRE [45°/45°]_4_	29.7 ± 0.2	4611.6 ± 74.1	11.9 ± 0.2	-	4728.7 ± 136	-
HCF-GFRE [45°/45°]_4_	29.1 ± 0.2	4456.8 ± 132	12.4 ± 0.3	-	4860.6 ± 185	-

## Data Availability

Data available in a publicly accessible repository.

## Abbreviations

CT	Computed Tomography
EPD	Energy Profile Diagram
GFRE	Glass Fibers Reinforced Elium
HCF	High Cycle Fatigue
LCF	Low Cyclic Fatigue
PMMA	Poly[Methyl Methacrylate]
SEM	Scanning Electron Microscopy
TGA	Thermogravimetric Analysis
